# Image-based 3D non-rigid respiratory motion correction for free-breathing thoracic MR angiography

**DOI:** 10.1186/1532-429X-18-S1-P15

**Published:** 2016-01-27

**Authors:** Fei Han, Cheng Ouyang, Ziwu Zhou, Paul J Finn, Peng Hu

**Affiliations:** 1 Radiology, UCLA, Los Angeles, CA USA; 2 Biomedical Engineering, Xi'an Jiaotong University, Xi'an, China

## Background

Respiratory motion compensation is usually required in free-breathing thoracic MR imaging applications to remove the motion induced blur and ghosting artifacts. Most respiratory motion compensation techniques are based on gating in which data is acquired only at one respiratory state (RS) and therefore suffer from extended yet unpredictable scan time. Other techniques compensate for motion by correcting the phase of motion corrupted k-space data so that data acquired in multiple respiratory states can be used in reconstruction for improved scan efficiency. However, these methods are often limited to correcting rigid-body motion and thus not suitable for thoracic imaging with large field-of-view. In this work, we propose a technical strategy of correcting 3D non-rigid motion and apply it to ferumoxytol enhanced free-breathing thoracic MRA.

## Methods

**Figure 1:**
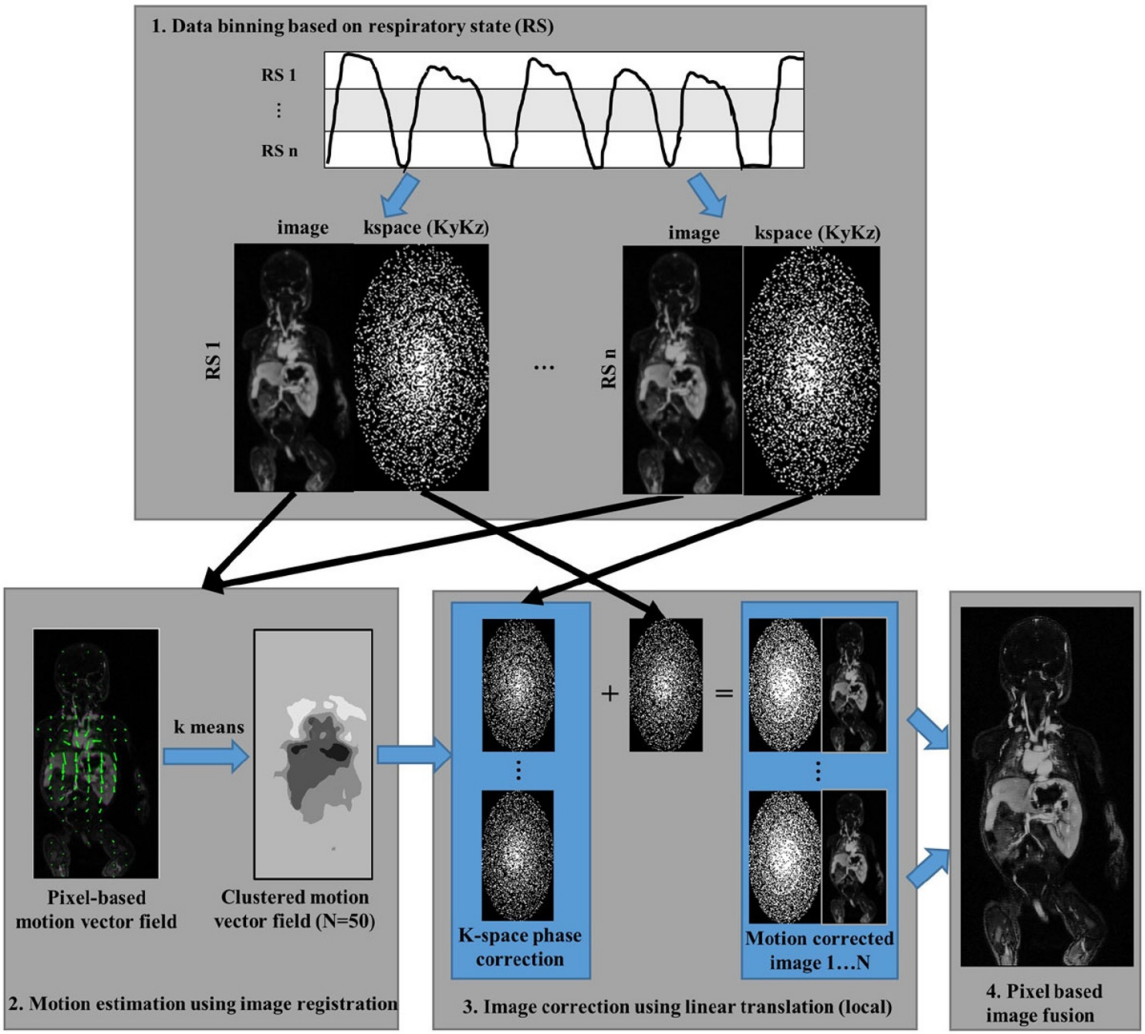
**Workflow of the proposed strategy**. Complex respiratory motion is modeled using voxel-based linear translations, which are estimated using 3D image registration after data binning using a respiratory surrogate signal. The voxel-based motion vector is represented by fewer approximations (N = 50) after k-means clustering for reduced computation. Linear k-space phase correction is performed for each motion vector and combined with reference bin for image reconstruction. The final output image is generated by pixel-based image fusion where the voxel value is selected from the motion corrected images (1..N) with the corresponding motion vector.

In the proposed method, the non-rigid motion is modeled using voxel-based linear translation, which is estimated using 3D image registration. The workflow is listed in Fig. 1: 1) acquired data is binned into different RS based on a respiratory surrogate signal (e.g. navigator, self-gating). Dynamic k-space sampling pattern is required so that images can be reconstructed from each data bin; 2) quantitative motion estimation is performed between a chosen "reference bin" and one of the "correction bins" using 3D non-rigid image registration, generating a voxel-based 3D motion vector field. Motion vectors are then processed using k-means clustering and represented by fewer approximations for reduced computational cost in the next step; 3) kspace linear phase corrections are performed using each motion vector on the "correction bin", which are then combined with the reference bin for reconstruction; 4) the final images are generated by pixel-based image fusion of all the motion corrected image candidate, where the selection is made based on the estimated motion vector in step 2.

The proposed method was applied on a ferumoxytol-enhanced free-breathing thoracic MRA dataset acquired on a 1 year old patient under general anesthesia with mechanical ventilation. The data was retrospectively binned into 2 RS using airway pressure signal. The kspace under-sampling rate of each bin was 12X. Other scan parameters includes: matrix = 500 × 240 × 120; 1mm^3^ resolution; 6 cardiac phases; TA = 6 min. Image registration was performed using Elastix toolbox. Images were reconstructed using ESPIRiT.

## Results

The motion corrected image has higher SNR (blood pool: 25.4 vs 14.5) than the ones reconstructed from single RS because double amount of kspace data was used. When compared with the images from uncorrected 2 RS, vascular and cardiac structures in the chest and abdomen are better defined in the motion corrected images. (Fig. 1)

## Conclusions

Our preliminary result shows that the proposed method could correct for complex respiratory motion in thoracic and abdomen imaging applications. The scan efficiency could be significantly increased since more k-space data is used.

**Figure 2:**
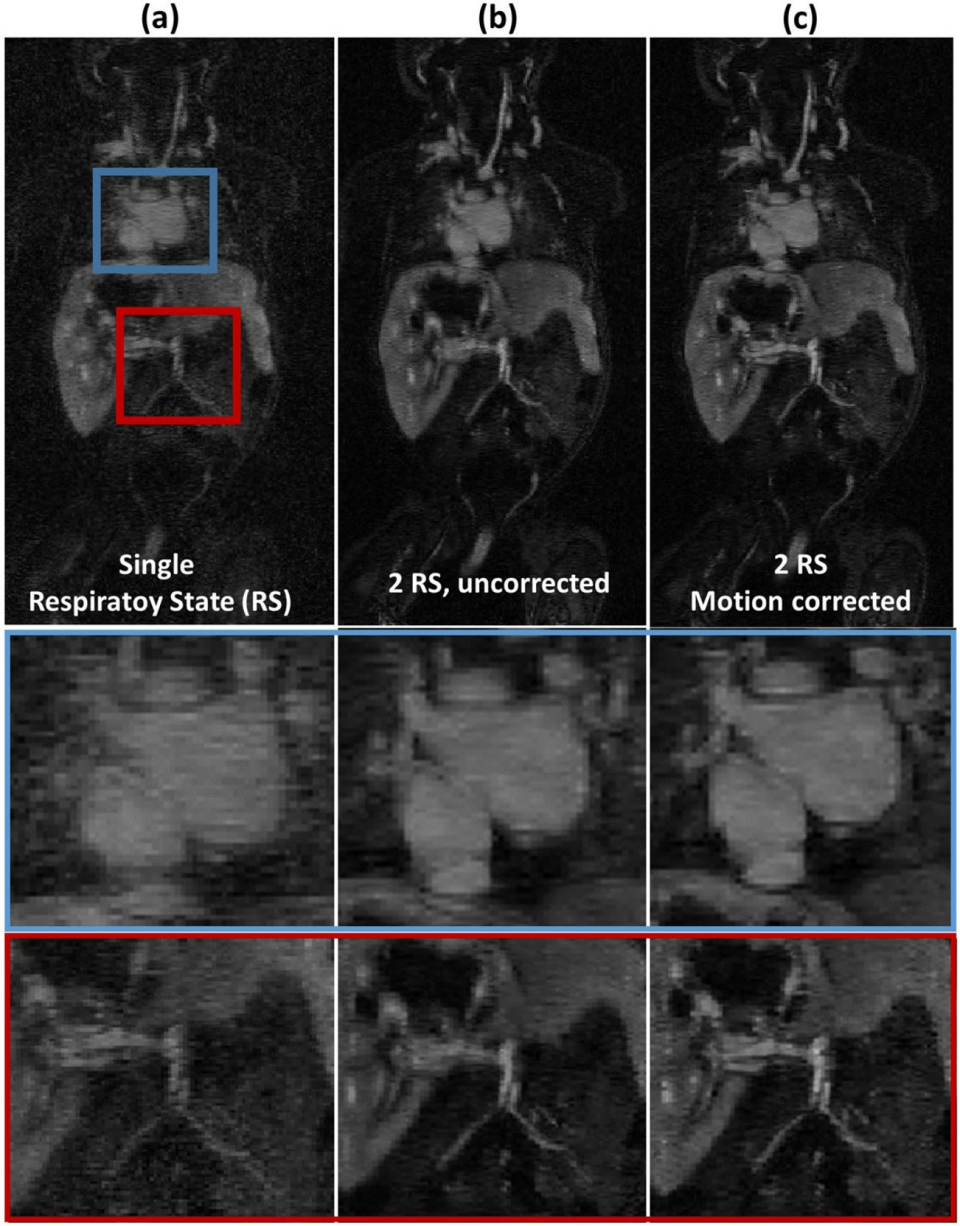
**Reconstructed ferumoxytol-enhanced non-breath-held angiography images using data from single respiratory state (a), two respiratory states without correction (b) and two respiratory states with the non-rigid motion correction**. The images using 2 respiratory states (b,c) has higher SNR than (a) because the amount of kspace data used in reconstruction is doubled. The vascular and cardiac structures in different regions of the motion corrected image (c) are better defined than the ones in image without correction.

